# The Predictive Value of Machine Learning for Postoperative Delirium in Cardiac Surgery: Systematic Review and Meta-Analysis

**DOI:** 10.2196/72304

**Published:** 2026-02-23

**Authors:** Yi Guo, Hong Xu, Ankui Wang, Mingming Zhang, Shuai Zhang, Peng Xie

**Affiliations:** 1 Department of Anesthesiology, Norinco General Hospital, Xi'an, China; 2 Department of Operating Room, Norinco General Hospital, Xi'an, China; 3 Department of Hepatobiliary and Vascular Surgery, Norinco General Hospital, No. 12, Zhangba East Road, Yanta District, Xi'an, China, 86 17792723769

**Keywords:** cardiac surgery, delirium, machine learning, predictive model, systematic review

## Abstract

**Background:**

Postoperative delirium (POD) following cardiac surgery is a severe complication, and early identification of delirium risk remains a challenge in clinical practice. While machine learning (ML) has garnered increasing attention in health care applications, effective early prediction tools remain limited in current clinical practice. Recent investigations have explored the effectiveness of ML-based methods for identifying the risk of POD in patients undergoing cardiac surgery. However, more evidence is required to validate the feasibility of these methods.

**Objectives:**

This study aims to ascertain the performance of ML in identifying the risk of POD following cardiac surgery, providing evidence for the development or updating of future ML-based assessment tools.

**Methods:**

A comprehensive literature search was conducted across 4 databases—PubMed, the Cochrane Library, Embase, and Web of Science—through August 30, 2024, to identify studies investigating individual POD risk prediction using ML approaches and nomograms. The risk of bias of the models in the included studies was assessed leveraging the Prediction Model Bias Risk Assessment Tool. Subgroup analyses were performed based on datasets, validation methods, study types, risk of bias, and model types.

**Results:**

The analysis incorporated 28 original studies comprising 80,143 patients undergoing cardiac surgery, of whom 6326 developed POD. Meta-analysis revealed that, in validation datasets, the c-index, sensitivity, and specificity for delirium prediction reached 0.805 (95% CI 0.759‐0.852), 0.72 (95% CI 0.65‐0.79), and 0.78 (95% CI 0.71‐0.83), respectively. Logistic regression was the primary modeling method. In validation datasets, the c-index, sensitivity, and specificity reached 0.773 (95% CI 0.724‐0.823), 0.73 (95% CI 0.64‐0.80), and 0.70 (95% CI 0.65‐0.74), respectively.

**Conclusions:**

ML-based prediction tools for POD following cardiac surgery demonstrate promising performance. However, the limited number of studies and validation approaches necessitate cautious interpretation of these findings. Future multicenter studies are warranted to develop more robust ML-based prediction tools, enabling precise risk stratification and targeted preventive interventions for POD.

## Introduction

The number of deaths from cardiovascular disease far exceeds those from cancer [1], and cardiac surgery is often required for its treatment. Common types of cardiac surgery include congenital heart disease surgery, coronary artery bypass grafting, cardiac valve surgery, and ascending aorta and arch surgery. Although cardiac surgery has benefitted a large number of patients, postoperative complications remain a significant clinical challenge. Common complications include excessive bleeding, refractory shock, postoperative cardiac arrest, neurologic injury, respiratory failure, and acute respiratory distress syndrome [2], as well as gastrointestinal complications [3]. Delirium, characterized by acute changes in mental status with confusion and impaired attention, is one of the most common and serious neurological complications following cardiac surgery, with reported incidence rates exceeding 50% [4,5]. A study by Salluh et al [6] found that delirium correlates with higher in-hospital mortality rates, extended hospital stays, and postdischarge cognitive impairment. Therefore, early prediction of delirium risk and the development of effective, targeted preventive strategies are of considerable clinical importance.

Currently, there is no universally recognized or effective tool for predicting postoperative delirium (POD) in cardiac surgery. Machine learning (ML) can integrate high-dimensional data to develop and validate clinical prediction tools for disease prediction, diagnosis, treatment response, prognosis, and adverse events. Several researchers have constructed predictive models for POD using ML techniques [7-9]; however, the accuracy of these models requires further validation. In the Shining Cai study [7], prediction models for postcardiac surgery delirium were summarized, but the number of included studies was limited, possibly due to the search strategy. Both Lee et al [8] and Chen et al [9] focused on POD prediction models and demonstrated promising predictive value. However, the study by Lee et al [8] specifically focused on delirium occurring in the intensive care unit after cardiac surgery, whereas Chen et al [9] examined POD without specifying cardiac surgery. Therefore, this study aims to evaluate the effectiveness of ML-based predictive models for delirium after cardiac surgery and to inform the future development or refinement of simplified clinical scoring tools.

## Methods

### Study Registration

This study adhered to the PRISMA (Preferred Reporting Items for Systematic Reviews and Meta-Analyses) 2020 guidelines and was registered with PROSPERO (registration number CRD42024588522).

### Inclusion and Exclusion Criteria

Eligibility criteria were defined before literature screening (Table 1).

**Table 1.** Eligibility criteria for original studies included in this systematic review.

Items	Inclusion criteria	Exclusion criteria
P (Population)	Patients who underwent cardiac surgery, including CABG^a^, OPCABG^b^, valve surgery, ascending aorta and arch surgery, and minimally invasive procedures such as TAVR^c^	Studies that did not clearly distinguish cardiac surgery from other surgical procedures
I (Intervention)	Studies that developed a complete predictive model for POD^d^ after cardiac surgery	Studies limited to risk factor identification without a complete ML^e^ model, or studies that assessed only univariate predictive accuracy
C (Control)	None	None
O (Outcomes)	Evaluation of ML model performance using metrics such as c-index, sensitivity (Sen), specificity (Spe), accuracy, precision, diagnostic contingency tables, or *F*_1_-score	Studies lacking outcome measures for model accuracy
S (Study design)	Case-control, cohort, or cross-sectional studies published in English	Studies evaluating only established scales (for studies using overlapping datasets, only the study with the largest sample size was retained)

a CABG: coronary artery bypass graft.

b OPCABG: off-pump coronary artery bypass grafting.

c TAVR: transcatheter aortic valve replacement.

d POD: postoperative delirium.

e ML: machine learning.

### Data Sources and Search Strategy

A comprehensive literature search was executed in 4 databases: PubMed, the Cochrane Library, Embase, and Web of Science, through August 30, 2024. Both Medical Subject Headings and free-text terms were used for the search. No restrictions were imposed on regions and year of publication (Table S1 in Multimedia Appendix 1).

### Study Selection and Data Extraction

The retrieved records were imported into EndNote, and after removing duplicates, titles and abstracts were reviewed. Full texts of potentially relevant articles were obtained and checked. To reduce population heterogeneity, this review focused exclusively on cardiac surgery. Because some relevant studies did not explicitly mention cardiac surgery in the title or abstract, the term “cardiac surgery” was not included as a search keyword; instead, noncardiac surgical studies were excluded manually during the screening process.

A standardized digital form was leveraged for data collection. Extracted information included title, DOI, authors, year of publication, study design, patient source, type of cardiac surgery, diagnostic criteria for delirium, number of patients with delirium, total number of cardiac surgery patients, number of delirium patients in both training and validation sets, validation set formation method, total number of patients in the validation set, missing data and methods for handling missing data, variable screening methods, modeling methods, and predictive factors.

Two researchers independently reviewed the literature and cross-checked the extracted data, with a third investigator resolving any disagreements.

### Risk of Bias in Included Studies

Risk of bias in the included studies was appraised using the Prediction Model Risk of Bias Assessment Tool (PROBAST) [10], which included questions across domains including participants, predictors, outcomes, and statistical analysis. These domains reflected the overall risk of bias and applicability of the studies. Specific questions within domains had 3 possible responses: yes/likely, no/likely not, and no information. If the risk of bias in all domains was low for a study, the study was marked as low risk. If the risk of bias in at least a domain was high, the study was graded to have a high risk of bias. Two researchers independently performed and cross-checked the risk of bias assessments, with a third researcher resolving any disagreements.

### Statistical Analysis

A meta-analysis was performed to evaluate the overall performance of ML models using the c-index. For studies lacking CIs or SEs of c-index, SEs were estimated according to the study by Debray et al [11]. The heterogeneity between studies was quantified by leveraging the *I*² statistic. If *I*² was greater than 50%, a random-effects model was utilized. If *I*² was less than 50%, a fixed-effects model was applied. Funnel plots were employed to detect publication bias of the c-index. The Egger test was applied for statistical assessment of publication bias.

A bivariate mixed-effects model was used to synthesize sensitivity and specificity data. For studies without diagnostic contingency tables, these were reconstructed using sensitivity, specificity, precision, and case numbers for calculations. Subgroup analyses were performed based on validation methods, study types, risk of bias, and model types. The statistical computation was conducted using Stata version 15.0.

## Results

### Study Selection

**Figure 1.**
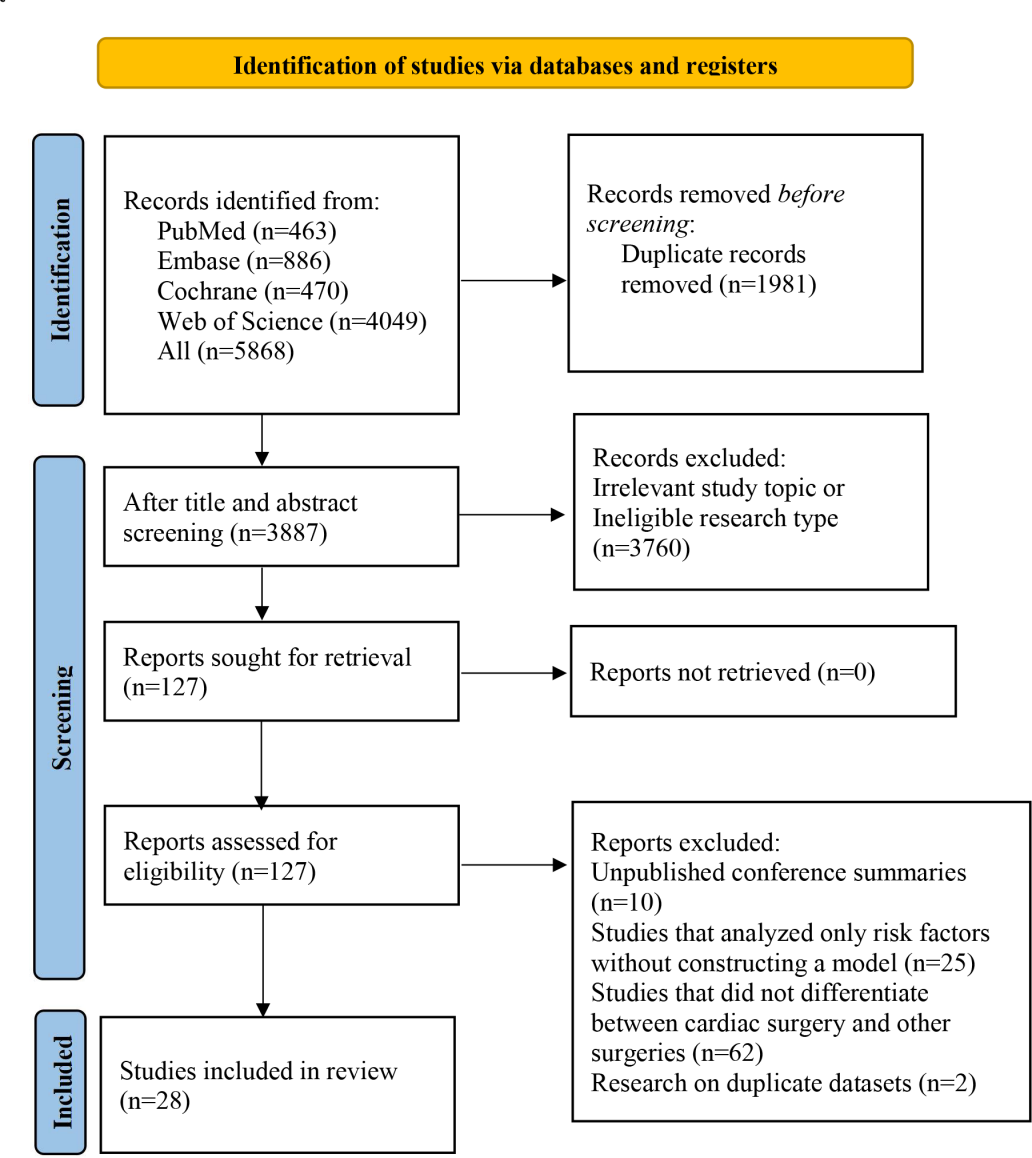
PRISMA (Preferred Reporting Items for Systematic Reviews and Meta-Analyses) flow diagram of the study selection process.

The initial search yielded 5868 articles. After the removal of 1981 duplicates, 3760 studies were excluded based on titles and abstracts because they were unrelated to the research theme or inconsistent with the original study design. A total of 127 studies underwent full-text review. Of these, 10 unpublished conference abstracts, 25 studies limited to risk factor analysis without construction of a complete prediction model, 62 studies that failed to clearly distinguish cardiac surgery from noncardiac surgery, and 2 studies with overlapping datasets were excluded. Eventually, 28 studies [12-39] were included in the final analysis (Figure 1).

### Study Characteristics

The analysis included 28 publications from 2012 to 2024. The studies collectively involved 80,143 patients undergoing cardiac surgery, of whom 6326 developed delirium. These studies included 12 retrospective cohort studies, 14 prospective cohort studies, and 2 retrospective (for model development) and prospective (for model validation) cohort studies. These studies spanned 12 countries, with 13 studies originating from China. Five studies were multicenter, one was based on a registry database, and 23 studies were single-center.

The studies included coronary artery bypass grafting, valve surgery, congenital heart surgery (patients aged ≤8 y), cardiac tumor resection, aortic arch repairs, and type A aortic dissection treated with open-heart surgery. Most studies described multiple types of cardiac surgery, whereas only a few studies focused on a single surgery type. All 28 studies clearly outlined the diagnostic methods or steps for identifying delirium. To minimize population heterogeneity, the 2 pediatric studies and the remaining 26 adult studies were analyzed separately. Among the 26 adult studies, 21 included a validation set and described its generation method, comprising 1 external validation, 4 temporal validations, 11 random split validations, 3 k-fold cross-validations, and 2 bootstrap resampling validations. One study performed temporal validation but did not report validation set results due to inadequate sample size. To avoid sample size inflation, only the best-performing model from each study was selected, resulting in 9 model types, with logistic regression models being the most prevalent (Table S2 in Multimedia Appendix 2) [12-39].

In addition, the variables used for modeling were primarily clinical features. The modeling variables are presented in Table S3 in Multimedia Appendix 3.

### Risk of Bias Assessment

Risk of bias was assessed for the 28 models from the 28 studies. In the participant domain, some studies had only validated a single model, which contributed to potential bias. Twelve models were from retrospective cohort studies, resulting in a high risk of bias in participant selection. Given that all studies constructed models based on clinical features, the retrospective cohort models may introduce bias in assessing clinical characteristics, thereby increasing the risk of bias in the prediction factors as well. All included studies used established diagnostic criteria for delirium, and no prediction factors that required delirium assessment were incorporated into the modeling process, indicating low bias risk in outcome assessment.

In the predictors domain, only one study was rated as high risk of bias due to the incorporation of outcome information during predictor assessment, whereas all other studies demonstrated low risk of bias.

In the outcomes domain, none of the studies explicitly reported blinding; consequently, all models were rated as having unclear risk of bias for this domain.

**Figure 2.**
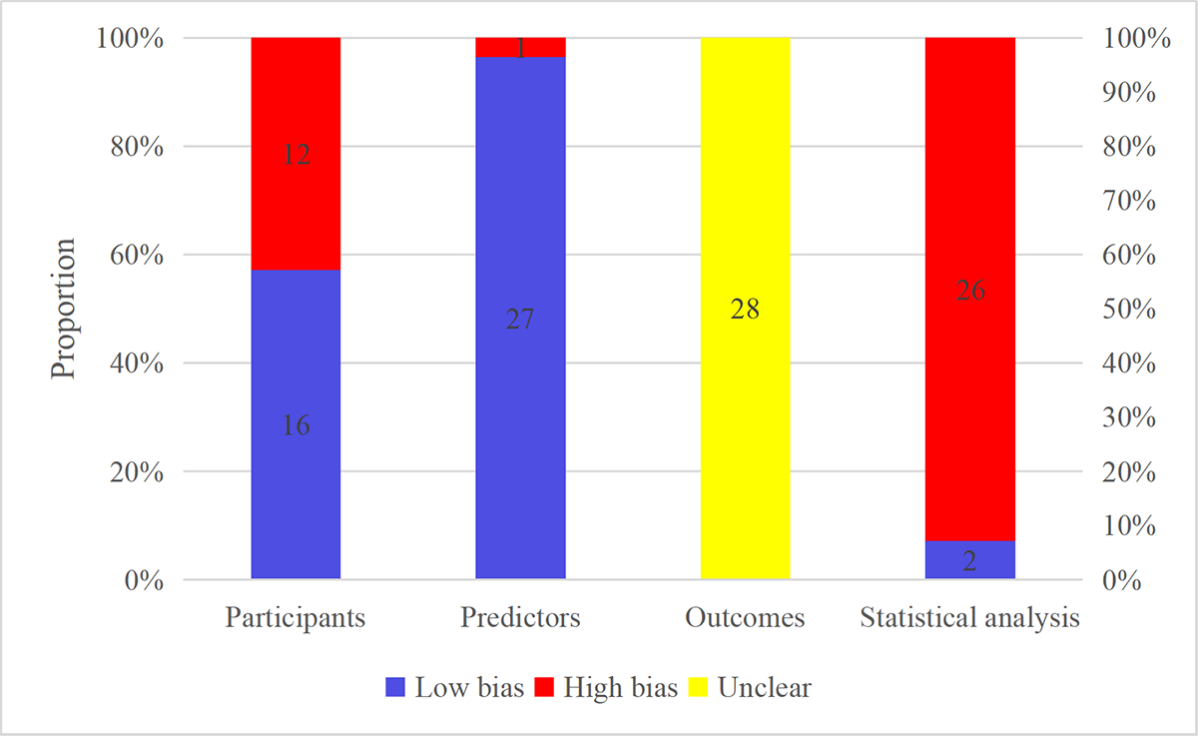
Risk of bias of the included predictive models assessed with the Prediction Model Risk of Bias Assessment Tool.

Fifteen models had a high risk of bias from insufficient events per variable (EPV <20) or lack of independent validation. Twenty-three models showed a high risk of bias in missing data handling, including direct deletion of missing values. Two studies showed unclear risk of bias due to inadequate description of predictor selection methods. Seventeen models demonstrated a high risk of bias for inadequate assessment of model fit or overfitting [40]. Overall, 26 models were identified as high-risk and 2 low-risk prediction models in statistical analysis (Figure 2).

### Meta-Analysis

#### C-Index

Because training set data were unavailable from 9 studies, only 17 prediction models for POD following cardiac surgery were reported in the training set. A random-effects model was leveraged, and the combined c-index was 0.815 (95% CI 0.754‐0.876). For validation, 20 models were reported for validating the predictive accuracy of POD following cardiac surgery. The pooled c-index, also derived from a random-effects model, was 0.805 (95% CI 0.759‐0.852) (Table 2).

**Table 2.** Meta-analysis results of c-index in the included studies for machine learning prediction of postoperative delirium following cardiac surgery in adults.

Subgroups	Training set			Validation set		
	Studies, n	c-index (95% CI)	*I*^2^ (%)	Studies, n	c-index (95% CI)	*I*^2^ (%)
Validation methods						
Random split validation	7	0.769 (0.692‐0.847)	97.6	11	0.791 (0.722‐0.860)	98.4
K-fold cross-validation	—^a^	—	—	3	0.845 (0.769‐0.921)	73.9
Bootstrap resampling validation	—	—	—	2	0.805 (0.745‐0.866)	47.1
External validation	1	0.740 (0.665‐0.815)	—	1	0.750 (0.667‐0.833)	—
Temporal validation	4	0.932 (0.895‐0.969)	94.2	3	0.848 (0.807‐0.889)	16.8
No validation	5	0.801 (0.744‐0.857)	74.8	—	—	—
Study types						
Retrospective cohort study	6	0.819 (0.696‐0.943)	99.5	10	0.803 (0.734‐0.871)	98.4
Prospective cohort study	9	0.809 (0.758‐0.860)	88.7	8	0.817 (0.762‐0.872)	82.6
Retrospective and prospective cohort study	2	0.832 (0.599‐1.065)	98.7	2	0.789 (0.621‐0.958)	93.8
Risk of bias (statistic analysis)						
Low	1	0.740 (0.665‐0.815)	—	1	0.750 (0.667‐0.833)	—
High	16	0.819 (0.756‐0.882)	98.9	19	0.808 (0.761‐0.856)	97.4
Model types						
ANN^b^	2	0.867 (0.744‐0.989)	98.5	2	0.781 (0.742‐0.820)	0
DT^c^	—	—	—	1	0.930 (0.885‐0.975)	—
KNN^d^	—	—	—	1	0.901 (0.866‐0.935)	—
LASSO^e^	1	0.741 (0.678‐0.804)	—	1	0.640 (0.531‐0.749)	—
LR^f^	13	0.801 (0.715‐0.887)	98.9	11	0.773 (0.724‐0.823)	91.1
RF^g^	—	—	—	1	0.920 (0.915‐0.925)	—
SVM^h^	—	—	—	1	0.941 (0.919‐0.963)	—
XGBoost^i^	—	—	—	1	0.760 (0.651‐0.869)	—
LGBM^j^	1	0.950 (0.935‐0.965)	—	1	0.877 (0.808‐0.946)	—
Overall	17	0.815 (0.754‐0.876)	98.8	20	0.805 (0.759‐0.852)	97.3

a Not applicable.

b ANN**: **artificial neural network.

c DT: decision tree.

d KNN: k-nearest neighbor.

e LASSO: least absolute shrinkage and selection operator.

f LR: logistic regression.

g RF: random forest.

h SVM: support vector machine.

i XGBoost: extreme gradient boosting.

j LightGBM: light gradient boosting machine.

Subgroup analysis results are also presented in Table 2. Of note, Subgroup analysis by model type indicated that most models were based on logistic regression. This approach achieved a c-index of 0.801 (95% CI 0.715‐0.887) in the training set and 0.773 (95% CI 0.724‐0.823) in the validation set. These values were lower than the overall c-index, suggesting that the performance of logistic regression models may be less effective than non-logistic regression models.

Notably, subgroup analyses revealed that heterogeneity remained extremely high across most subgroups and overall (*I*²>90%), indicating substantial unexplained variability among performance estimates. This extreme heterogeneity suggests that while pooled c-indices were calculated, their clinical interpretability is limited; these values should be considered a broad approximation of possible outcomes rather than precise summary effect estimates.

#### Sensitivity and Specificity

In the subgroup analysis by model types, diagnostic 2 × 2 tables from 13 models were available in the training set, and 18 models were available in the validation set. A bivariate random-effects model was leveraged. The majority of models were based on logistic regression, with pooled sensitivity and specificity of 0.75 (95% CI 0.62‐0.85) and 0.79 (95% CI 0.72‐0.85) in the training set, and 0.73 (95% CI 0.64‐0.80) and 0.70 (95% CI 0.65‐0.74) in the validation set. Sensitivity and specificity results for other subgroups are presented in Table 3.

**Table 3.** Meta-analysis results of sensitivity and specificity in the included studies for machine learning prediction of postoperative delirium following cardiac surgery in adults.^a^

Subgroups	Training set			Validation set		
	Studies, n	Sensitivity (95% CI) or range	Specificity (95% CI) or range	Studies, n	Sensitivity (95% CI) or range	Specificity (95% CI) or range
Verification methods						
Random split validation	7	0.68 (0.56‐0.78)	0.77 (0.69‐0.83)	10	0.72 (0.62‐0.80)	0.76 (0.67‐0.82)
K-fold cross-validation	—^b^	—	—	3	0.67‐0.86	0.72‐0.91
Bootstrap resampling validation	—	—	—	2	0.71‐0.91	0.67‐0.68
External validation						
Temporal validation	3	0.72‐0.97	0.80‐0.93	3	0.35‐0.79	0.68‐0.96
No validation	3	0.66‐0.81	0.66‐0.82	—	—	—
Study types						
Retrospective cohort study	5	0.80 (0.52‐0.93)	0.82 (0.72‐0.89)	10	0.74 (0.66‐0.80)	0.77 (0.69‐0.84)
Prospective cohort study	7	0.76 (0.70‐0.82)	0.78 (0.72‐0.84)	6	0.77 (0.67‐0.85)	0.70 (0.65‐0.75)
Retrospective and prospective cohort study	1	0.64	0.64	2	0.35‐0.53	0.77‐0.96
Risk of bias (statistic analysis)						
Low	—	—	—	—	—	—
High	13	0.76 (0.66‐0.84)	0.79 (0.73‐0.84)	18	0.72 (0.65‐0.79)	0.78 (0.71‐0.83)
Model types						
ANN^c^	2	0.72‐0.91	0.72‐0.86	2	0.68‐0.73	0.73‐0.78
KNN^d^	—	—	—	1	0.67	0.91
LASSO^e^	1	0.72	0.73	1	0.60	0.62
LR^f^	10	0.75 (0.62‐0.85)	0.79 (0.72‐0.85)	10	0.73 (0.64‐0.80)	0.70 (0.65‐0.74)
RF^g^	—	—	—	1	0.86	0.92
SVM^h^	—	—	—	1	0.91	0.87
XGBoost^i^	—	—	—	1	0.67	0.79
LGBM^j^	—	—	—	1	0.35	0.96
Overall	13	0.76 (0.66‐0.84)	0.79 (0.73‐0.84)	18	0.72 (0.65‐0.79)	0.78 (0.71‐0.83)

a For subgroups with fewer than 4 studies (n<4), quantitative pooling was not performed; values presented represent the range from original studies. For subgroups with 1 study (n=1), the presented values are single numbers.

b Not applicable.

c ANN: artificial neural network.

d KNN: k-nearest neighbor.

e LASSO: least absolute shrinkage and selection operator.

f LR: logistic regression.

g RF: random forest.

h SVM: support vector machine.

i XGBoost: extreme gradient boosting.

j LightGBM: light gradient boosting machine.

The pooled sensitivity and specificity were 0.76 (95% CI 0.66‐0.84) and 0.79 (95% CI 0.73‐0.84) in the training set and 0.72 (95% CI 0.65‐0.79) and 0.78 (95% CI 0.71‐0.83) in the validation set, respectively (Table 3).

#### Publication Bias Analysis

**Figure 3.**
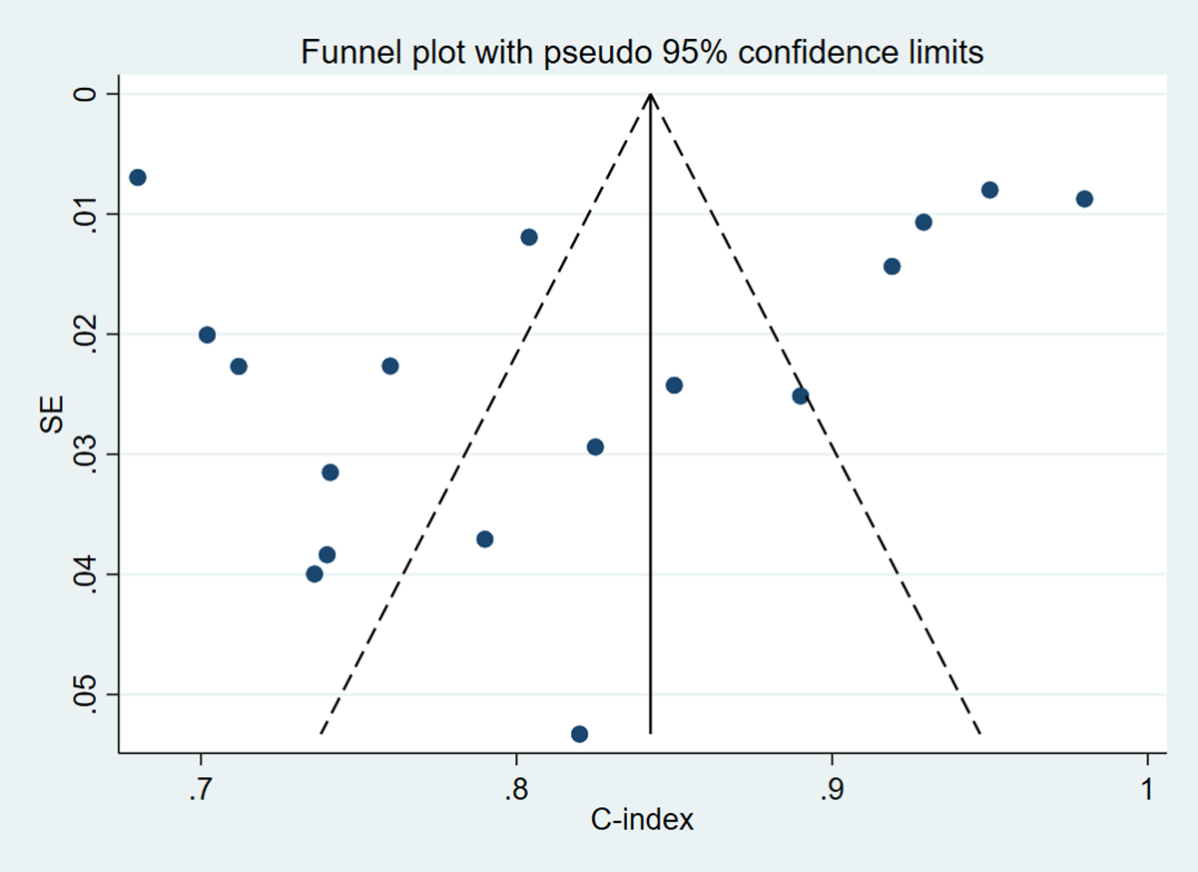
Publication bias analysis in the training set*.*

**Figure 4.**
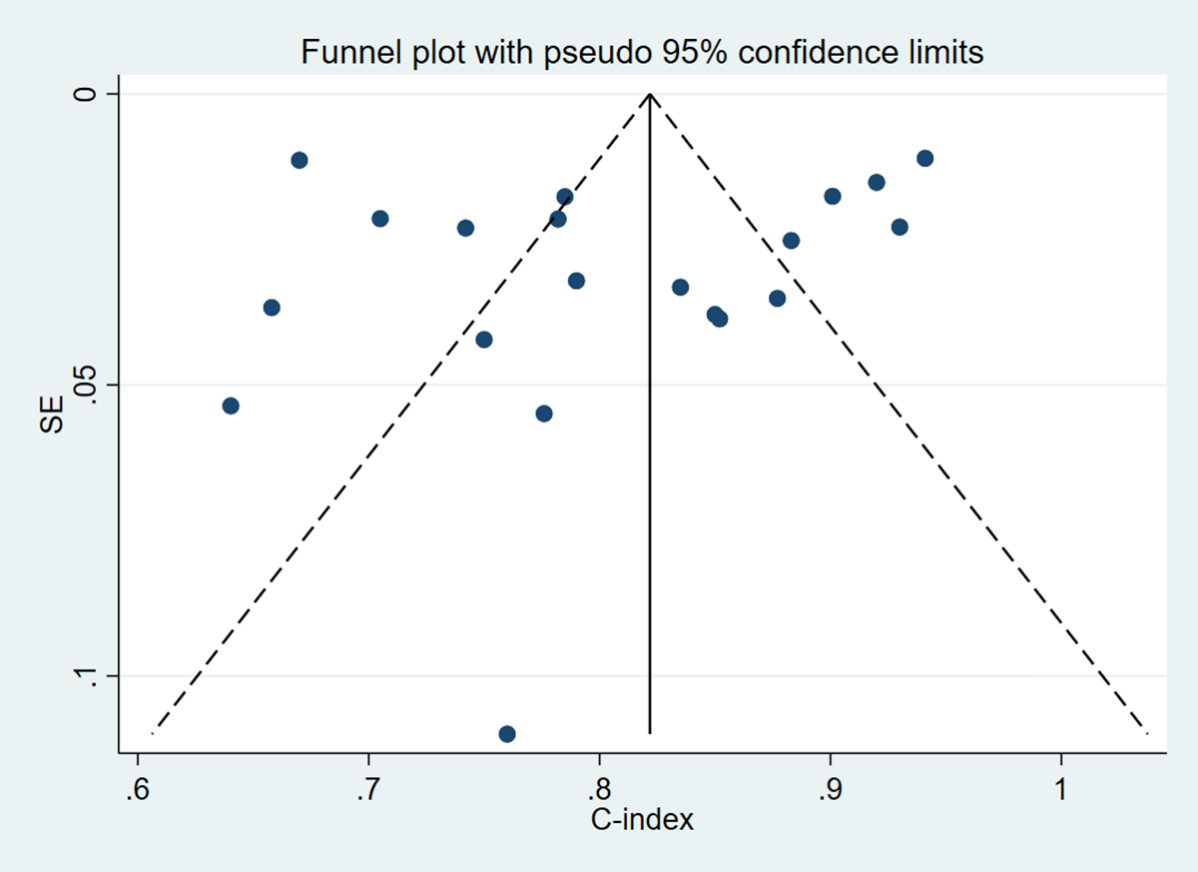
Publication bias analysis in the validation set*.*

Funnel plots were generated separately for the training and validation sets to assess publication bias. The results indicated no significant publication bias among the included studies in either the training (*P* value for Egger test was .65; Figure 3) or validation set (*P* value for Egger test was .59; Figure 4).

#### Studies for Prediction of POD Following Cardiac Surgery in Children

Two studies focusing on pediatric patients for predicting POD following cardiac surgery were ultimately included. Both were single-center studies that employed least absolute shrinkage and selection operator regression combined with multivariable logistic regression for variable selection and constructed nomogram models. The validation sets utilized random split validation and temporal validation, respectively. A meta-analysis was performed to synthesize model performance. In the validation set, the c-index, sensitivity, and specificity were 0.845 (95% CI 0.789‐0.900), 0.84, and 0.80, respectively.

## Discussion

### Main Findings

This study revealed growing research interest in early prediction of POD following cardiac surgery, with ML models demonstrating promising effectiveness. However, the majority of existing models are characterized by substantial heterogeneity and high risk of bias. Logistic regression remained the predominant modeling method, showing favorable performance.

### Comparison With Previous Reviews

Previous studies have reviewed early prediction methods for POD following cardiac surgery. For instance, a study by Cai et al [7] reviewed the use of ML in predicting POD in cardiac surgery. However, the included studies were limited, as the search strategy did not specifically account for the types of cardiac surgery. Furthermore, their study only provided the range of c-index values for the training set (0.74‐0.91) and external validation set (0.54‐0.90) without a quantitative systematic review. This limitation hindered a thorough interpretation of the value of ML in predicting POD in cardiac surgery.

In contrast, a study by Lee et al [8] summarized 3 well-established scoring tools for predicting POD in cardiac surgery. Their meta-analysis showed a pooled c-index of 0.62 (95% CI 0.58‐0.66), while the international recalibrated PREDELIRIC model demonstrated a c-index of 0.75 (95% CI 0.72‐0.79) in external validation. While these tools are well-established, they do not address the performance of ML. Our study has found that ML models appear to outperform these traditional scoring tools. Moreover, a review by Chen et al [9] examined POD in adults but did not specifically focus on cardiac surgery. The ML models included in their review demonstrated a c-index of 0.792 (95% CI 0.769‐0.816) for predicting POD following cardiac surgery. Our study, based on a more comprehensive body of evidence, systematically summarizes the predictive value of ML for POD following cardiac surgery, with results from validation sets demonstrating strong predictive performance.

Previous studies have reviewed the main factors predicting POD in cardiac surgery. Koster et al [41] identified predisposing risk factors such as atrial fibrillation, cognitive impairment, depression, stroke history, advanced age, and peripheral vascular disease. The frequency statistics in Table S3 in Multimedia Appendix 3 of the present study show that age, creatinine level, cardiopulmonary bypass duration, Mini-Mental State Examination score, and left ventricular ejection fraction are the most frequently used modeling variables in existing studies. The differences between the risk factors highlighted in this review and those reported in prior models may reflect variations in study design or data sources. Due to significant differences in modeling methods and population characteristics among the included studies, this review did not conduct formal predictive efficacy analysis to determine key predictive variables. The actual predictive value of these high-frequency variables requires further verification through more standardized designed studies.

### Challenges in Clinical Translation

The studies incorporated in this review involve diverse ML models, with some observed differences in the predictive performance of these models, prompting us to summarize their predictive value for POD following cardiac surgery. The study has found that logistic regression is currently the predominant predictive model, as it allows for the construction of widely applicable or simpler predictive nomograms. These nomograms provide excellent interpretability of the relationships between clinical indicators and delirium. Although the interpretability of predictive models is a crucial measure in clinical research, we found that the accuracy of logistic regression is lower than that of several other ML models. Other models are less interpretable, which hinders their further application. Additionally, literature on these other models remains relatively scarce. Therefore, future studies should develop ML models with potentially higher accuracy, enabling earlier identification of delirium risk in cardiac surgery patients and the implementation of preventive measures.

Among included studies, the PROBAST assessment revealed high risk of bias. In the 28 included studies, high risk of bias primarily stemmed from participant selection bias (eg, some studies did not explicitly exclude patients with severe neurological comorbidities), inadequate handling of missing data, and lack of external validation or reliance solely on internal validation with small samples. These methodological limitations impose certain constraints on the interpretation of our findings: although the pooled results reflect the overall trends observed across existing studies, the actual clinical reliability of ML models warrants cautious evaluation due to methodological deficiencies in the original studies. These findings also offer important implications for future research: subsequent studies should rigorously adhere to the PROBAST assessment criteria and optimize study designs—such as refining participant selection processes, strengthening data quality control, and conducting multicenter external validation—to enhance the reliability and generalizability of ML models.

Although the predictive value of ML for POD following cardiac surgery was summarized and demonstrated relatively promising results, substantial heterogeneity cannot be ignored. This challenge is commonly faced by current meta-analyses on ML. To better explain heterogeneity, subgroup analyses were conducted based on data sets (training sets and validation sets), validation methods, study types, bias risk, and model types. Substantial heterogeneity remained among different subgroups. This heterogeneity may result from the diverse modeling variables in the model training sets. Even within the same type of model, differences may exist in model parameters and selected modeling variables. Medical conditions in different countries also seem to have potential impacts. Given these potential influences, a random-effects model was adopted for pooling. Overall, due to the impact of heterogeneity, these results require cautious interpretation. In clinical applications, comprehensive judgment based on specific population characteristics and diagnosis and treatment scenarios is necessary to avoid direct application.

### Limitations

This study has several limitations. First, despite comprehensive systematic searching, the limited number of included studies may restrict result interpretation, particularly for models based on few studies. Second, original studies did not differentiate between cardiac surgery types and delirium occurrence locations (ICU vs general wards), precluding more detailed analysis. Third, most studies utilized internal validation without independent external validation, potentially limiting generalizability. Fourth, due to language search barriers, only English-language studies were included, potentially introducing bias and limiting result interpretation. Fifth, included populations showed limitations regarding source, sample size, and modeling method standardization, creating clinical application challenges without effective model stratification analysis. Sixth, this study can only reflect the variable usage trends in existing studies and cannot directly infer the predictive importance of variables. This may also result in certain established risk factors (eg, gender) appearing less frequently than their recognized importance in general clinical research would suggest. Such patterns reflect the variable selection outcomes of the ML models employed rather than the biological significance of these factors and therefore warrant cautious interpretation. Seventh, inherent limitations in the included studies—predominantly single-center, retrospective designs lacking rigorous external validation—contributed to the high risk of bias in the models, suggesting that the pooled predictive performance may be overestimated. The generalizability of these models requires further validation through prospective, multicenter studies. Therefore, future research should standardize model construction methods, enhance model transparency, and reduce bias risk to broaden clinical application. Additionally, our search strategy primarily focused on “nomogram” and specific ML terminology, potentially missing studies that reported traditional prediction models using only terms such as “logistic regression” or “multivariate analysis,” which may have introduced search bias. Although “nomogram” is a common presentation format for logistic regression models, future systematic reviews should consider more comprehensive search terms.

### Conclusions

This systematic review suggests that ML-based prediction tools for POD following cardiac surgery appear to demonstrate promising performance. However, the majority of existing models carry high risk of bias and lack rigorous external validation, potentially resulting in overly optimistic performance estimates. Current evidence is substantially constrained by extreme heterogeneity and low methodological quality. Future research should therefore standardize ML model development workflows, prioritize prospective designs, adhere to prediction model reporting standards, conduct extensive external validation, and perform comparative model studies to establish the actual clinical utility and feasibility of these tools in real-world settings.

## Supplementary material

10.2196/72304 Multimedia Appendix 1 Literature search strategy.

10.2196/72304 Multimedia Appendix 2 Basic information of included studies.

10.2196/72304 Multimedia Appendix 3 Modeling variables in included studies.

10.2196/72304 Checklist 1 PRISMA 2020 checklist.

## Abbreviations

ML: machine learning
POD: postoperative delirium
PRISMA: Preferred Reporting Items for Systematic Reviews and Meta-Analyses
PROBAST: Prediction Model Risk of Bias Assessment Tool
