# Supplementary material for: The Predictive Value of Machine Learning for Postoperative Delirium in Cardiac Surgery: Systematic Review and Meta-Analysis
Source: J Med Internet Res. 2026 Feb 23;28:e72304. doi: 10.2196/72304 (PMC12928544; doi:10.2196/72304)
Supplement: Multimedia Appendix 1 [file jmir-v28-e72304-s001.docx]

# **Table S1 Literature search strategy**

**1.Pubmed**

| Search number | Query | Results |
| --- | --- | --- |
| #1 | "Machine Learning"[Mesh] | 74,247 |
| #2 | ((((((((((((((((((((((((((((((((((((machine learning[Title/Abstract]) OR (artificial intelligence[Title/Abstract])) OR (Transfer Learning[Title/Abstract])) OR (Deep learning[Title/Abstract])) OR (Ensemble Learning[Title/Abstract])) OR (prediction model[Title/Abstract])) OR (risk model[Title/Abstract])) OR (risk score[Title/Abstract])) OR (random forest[Title/Abstract])) OR (neural network[Title/Abstract])) OR (neural networks[Title/Abstract])) OR (CNN[Title/Abstract])) OR (K-Nearest Neighbor[Title/Abstract])) OR (Support vector machine[Title/Abstract])) OR (SVM[Title/Abstract])) OR (Gradient Boosting Machine[Title/Abstract])) OR (Nomogram[Title/Abstract])) OR (XGBoost[Title/Abstract])) OR (Adaboost[Title/Abstract])) OR (LightGBM[Title/Abstract])) OR (CatBoost[Title/Abstract])) OR (Gradient Boosting[Title/Abstract])) OR (Decision tree[Title/Abstract])) OR (Regression Trees[Title/Abstract])) OR (ResNet[Title/Abstract])) OR (AlexNet[Title/Abstract])) OR (VGGNet[Title/Abstract])) ) OR (GoogLeNet[Title/Abstract])) OR (Naive Bayesian[Title/Abstract])) OR (Multilayer perceptron[Title/Abstract])) OR (Bayesian network[Title/Abstract])) OR (Radiomics[Title/Abstract])) OR (Radiomic[Title/Abstract])) OR (radiomics-based[Title/Abstract])) OR (radiomic signature[Title/Abstract])) OR (Texture[Title/Abstract]) | 442,169 |
| #3 | ("Machine Learning"[Mesh]) OR (((((((((((((((((((((((((((((((((((((machine learning[Title/Abstract]) OR (artificial intelligence[Title/Abstract])) OR (Transfer Learning[Title/Abstract])) OR (Deep learning[Title/Abstract])) OR (Ensemble Learning[Title/Abstract])) OR (prediction model[Title/Abstract])) OR (risk model[Title/Abstract])) OR (risk score[Title/Abstract])) OR (random forest[Title/Abstract])) OR (neural network[Title/Abstract])) OR (neural networks[Title/Abstract])) OR (CNN[Title/Abstract])) OR (K-Nearest Neighbor[Title/Abstract])) OR (Support vector machine[Title/Abstract])) OR (SVM[Title/Abstract])) OR (Gradient Boosting Machine[Title/Abstract])) OR (Nomogram[Title/Abstract])) OR (XGBoost[Title/Abstract])) OR (Adaboost[Title/Abstract])) OR (LightGBM[Title/Abstract])) OR (CatBoost[Title/Abstract])) OR (Gradient Boosting[Title/Abstract])) OR (Decision tree[Title/Abstract])) OR (Regression Trees[Title/Abstract])) OR (ResNet[Title/Abstract])) OR (AlexNet[Title/Abstract])) OR (VGGNet[Title/Abstract])) ) OR (GoogLeNet[Title/Abstract])) OR (Naive Bayesian[Title/Abstract])) OR (Multilayer perceptron[Title/Abstract])) OR (Bayesian network[Title/Abstract])) OR (Radiomics[Title/Abstract])) OR (Radiomic[Title/Abstract])) OR (radiomics-based[Title/Abstract])) OR (radiomic signature[Title/Abstract])) OR (Texture[Title/Abstract])) | 447,575 |
| #4 | Delirium[MeSH Terms] | 13,628 |
| #5 | ((((Delirium[Title/Abstract]) OR (Deliriums[Title/Abstract])) OR (delirious manifestation[Title/Abstract])) OR (delirious state[Title/Abstract])) OR (delirious syndrome[Title/Abstract]) | 23,049 |
| #6 | (Delirium[MeSH Terms]) OR (((((Delirium[Title/Abstract]) OR (Deliriums[Title/Abstract])) OR (delirious manifestation[Title/Abstract])) OR (delirious state[Title/Abstract])) OR (delirious syndrome[Title/Abstract])) | 25,050 |
| #7 | (("Machine Learning"[Mesh]) OR (((((((((((((((((((((((((((((((((((((machine learning[Title/Abstract]) OR (artificial intelligence[Title/Abstract])) OR (Transfer Learning[Title/Abstract])) OR (Deep learning[Title/Abstract])) OR (Ensemble Learning[Title/Abstract])) OR (prediction model[Title/Abstract])) OR (risk model[Title/Abstract])) OR (risk score[Title/Abstract])) OR (random forest[Title/Abstract])) OR (neural network[Title/Abstract])) OR (neural networks[Title/Abstract])) OR (CNN[Title/Abstract])) OR (K-Nearest Neighbor[Title/Abstract])) OR (Support vector machine[Title/Abstract])) OR (SVM[Title/Abstract])) OR (Gradient Boosting Machine[Title/Abstract])) OR (Nomogram[Title/Abstract])) OR (XGBoost[Title/Abstract])) OR (Adaboost[Title/Abstract])) OR (LightGBM[Title/Abstract])) OR (CatBoost[Title/Abstract])) OR (Gradient Boosting[Title/Abstract])) OR (Decision tree[Title/Abstract])) OR (Regression Trees[Title/Abstract])) OR (ResNet[Title/Abstract])) OR (AlexNet[Title/Abstract])) OR (VGGNet[Title/Abstract])) ) OR (GoogLeNet[Title/Abstract])) OR (Naive Bayesian[Title/Abstract])) OR (Multilayer perceptron[Title/Abstract])) OR (Bayesian network[Title/Abstract])) OR (Radiomics[Title/Abstract])) OR (Radiomic[Title/Abstract])) OR (radiomics-based[Title/Abstract])) OR (radiomic signature[Title/Abstract])) OR (Texture[Title/Abstract]))) AND ((Delirium[MeSH Terms]) OR (((((Delirium[Title/Abstract]) OR (Deliriums[Title/Abstract])) OR (delirious manifestation[Title/Abstract])) OR (delirious state[Title/Abstract])) OR (delirious syndrome[Title/Abstract]))) | 463 |

**2.Cochrane**

| Search number | Query | Results |
| --- | --- | --- |
| #1 | MeSH descriptor: [Machine Learning] explode all trees | 1002 |
| #2 | (machine learning):ti,ab,kw OR (artificial intelligence):ti,ab,kw OR (Transfer Learning):ti,ab,kw OR (Deep learning):ti,ab,kw OR (Ensemble Learning):ti,ab,kw | 7685 |
| #3 | (prediction model):ti,ab,kw OR (risk model):ti,ab,kw OR (risk score):ti,ab,kw OR (random forest):ti,ab,kw OR (neural network):ti,ab,kw | 74401 |
| #4 | (neural networks):ti,ab,kw OR (CNN):ti,ab,kw OR (K-Nearest Neighbor):ti,ab,kw OR (Support vector machine):ti,ab,kw OR (SVM):ti,ab,kw | 2700 |
| #5 | (Gradient Boosting Machine):ti,ab,kw OR (Nomogram):ti,ab,kw OR (XGBoost):ti,ab,kw OR (Adaboost):ti,ab,kw OR (LightGBM):ti,ab,kw | 2097 |
| #6 | (CatBoost):ti,ab,kw OR (Gradient Boosting):ti,ab,kw OR (Decision tree):ti,ab,kw OR (Regression Trees):ti,ab,kw OR (ResNet):ti,ab,kw | 1338 |
| #7 | (AlexNet):ti,ab,kw OR (VGGNet):ti,ab,kw OR (GoogLeNet):ti,ab,kw OR (Naive Bayesian):ti,ab,kw OR (Multilayer perceptron):ti,ab,kw | 205 |
| #8 | (Bayesian network):ti,ab,kw OR (Radiomics):ti,ab,kw OR (Radiomic):ti,ab,kw OR (radiomics-based):ti,ab,kw OR (radiomic signature):ti,ab,kw | 1118 |
| #9 | (Texture):ti,ab,kw | 2194 |
| #10 | #1 or #2 or #3 or #4 or #5 or #6 or #7 or #8 or #9 | 84497 |
| #11 | MeSH descriptor: [Delirium] explode all trees | 1613 |
| #12 | (Delirium):ti,ab,kw OR (Deliriums):ti,ab,kw OR (delirious manifestation):ti,ab,kw OR (delirious state):ti,ab,kw OR (delirious syndrome):ti,ab,kw | 6201 |
| #13 | #11 or #12 | 6201 |
| #14 | #10 and #13 | 471 |

**3.Embase**

| Search number | Query | Results |
| --- | --- | --- |
| #1 | 'machine learning'/exp | 504472 |
| #2 | 'machine learning':ab,ti OR 'artificial intelligence':ab,ti OR 'transfer learning':ab,ti OR 'deep learning':ab,ti OR 'ensemble learning':ab,ti OR 'prediction model':ab,ti OR 'risk model':ab,ti OR 'risk score':ab,ti OR 'random forest':ab,ti OR 'neural network':ab,ti OR 'neural networks':ab,ti OR cnn:ab,ti OR 'k-nearest neighbor':ab,ti OR 'support vector machine':ab,ti OR svm:ab,ti OR 'gradient boosting machine':ab,ti OR nomogram:ab,ti OR xgboost:ab,ti OR adaboost:ab,ti OR lightgbm:ab,ti OR catboost:ab,ti OR 'gradient boosting':ab,ti OR 'decision tree':ab,ti OR 'regression trees':ab,ti OR resnet:ab,ti OR alexnet:ab,ti OR vggnet:ab,ti OR googlenet:ab,ti OR 'naive bayesian':ab,ti OR 'multilayer perceptron':ab,ti OR 'bayesian network':ab,ti OR radiomics:ab,ti OR radiomic:ab,ti OR 'radiomics based':ab,ti OR 'radiomic signature':ab,ti OR texture:ab,ti | 578612 |
| #3 | #1 OR #2 | 817863 |
| #4 | 'delirium'/exp | 46114 |
| #5 | delirium:ab,ti OR deliriums:ab,ti OR 'delirious manifestation':ab,ti OR 'delirious state':ab,ti OR 'delirious syndrome':ab,ti | 34642 |
| #6 | #4 OR #5 | 51700 |
| #7 | #3 AND #6 | 886 |

**4.Web of science**

| Search number | Query | Results |
| --- | --- | --- |
| #1 | machine learning (Topic) OR artificial intelligence (Topic) OR Transfer Learning (Topic) OR Deep learning (Topic) OR Ensemble Learning (Topic) OR prediction model (Topic) OR risk model (Topic) OR risk score (Topic) OR random forest (Topic) OR neural network (Topic) OR neural networks (Topic) OR CNN (Topic) OR K-Nearest Neighbor (Topic) OR Support vector machine (Topic) OR SVM (Topic) OR Gradient Boosting Machine (Topic) OR Nomogram (Topic) OR XGBoost (Topic) OR Adaboost (Topic) OR LightGBM (Topic) OR CatBoost (Topic) OR Gradient Boosting (Topic) OR Decision tree (Topic) OR Regression Trees (Topic) OR ResNet (Topic) OR AlexNet (Topic) OR VGGNet (Topic) OR GoogLeNet (Topic) OR Naive Bayesian (Topic) OR Multilayer perceptron (Topic) OR Bayesian network (Topic) OR Radiomics (Topic) OR Radiomic (Topic) OR radiomics-based (Topic) OR radiomic signature (Topic) OR Texture (Topic) | 3651401 |
| #2 | Delirium (Topic) OR Deliriums (Topic) OR delirious manifestation (Topic) OR delirious state (Topic) OR delirious syndrome (Topic) | 26813 |
| #3 | #1 AND #2 | 4049 |
