# Supplementary material for: The Predictive Value of Machine Learning for Postoperative Delirium in Cardiac Surgery: Systematic Review and Meta-Analysis
Source: J Med Internet Res. 2026 Feb 23;28:e72304. doi: 10.2196/72304 (PMC12928544; doi:10.2196/72304)
Supplement: Multimedia Appendix 2 [file jmir-v28-e72304-s002.docx]

**Table S2** Basic Information of Included Studies.

| **No.** | **First author** | **Year of publication** | **Country of author** | **Populations** | **Study types** | **Patient source** | **Surgery types** | **Diagnostic criteria for delirium** | **Number of delirium cases** | **Total number of cases** | **Generation method of validation set** | **Number of cases in validation set** | **Modeling variables** | **Model type** |
| --- | --- | --- | --- | --- | --- | --- | --- | --- | --- | --- | --- | --- | --- | --- |
| 1 | Yutaka Hatano[1] | 2013 | Japan | Adults | Retrospective Cohort Study | Single center | CABG: coronary artery bypass surgery; OPCAB: Off-pump coronary artery bypass surgery; valve with or without CABG | The Diagnostic and Statistical Manual of Mental Disorders, Fourth Edition | 18 | 130 | None^1^ | None | Creatinine>1.1 mg/dL, Severe Deep White-Matter Hyperintensities(DWMH)(Fazekas score=3), Duration of surgery | LR |
| 2 | - Dou Mao[2] | 2023 | China | Children | Prospective Cohort Study | Single center | congenital heart surgery | The Chinese version of Cornell Assessment of Pediatric Delirium (CAPD) | 120 | 470 | Random Split Validation | 140 | Age, Disease severity, Non-invasive ventilation after extubation, Delayed chest closure, Phenobarbital dosage, Promethazine dosage, Use of mannitol, Elevated temperature | LR |
| 3 | - Changho Han[3] | 2024 | Republic of Korea | Adults | Retrospective(for model development)and Prospective (for model validation) Cohort Study | Single center | No CPB, CPB no TCA, CPB TCA (CPB, cardiopulmonary bypass; TCA, total circulatory arrest) | ICDSC (Intensive Care Delirium Screening Checklist), Screening Scale for Critical Care Delirium | 260 | 2114 | Temporal validation | 202 | estimated glomerular filtration rate (eGFR), age, T3, duration of BIS<25, Katz grade 4;duration of BIS<40 or PSI<25, average CI, AUC of PP<60 mmHg, duration of SR>1%, CV of rSO2, average rSO2, and ARV of mean arterial pressure (MAP) | LGBM |
| 4 | Xiuxiu Zhao[4] | 2023 | China | Adults | Retrospective Cohort Study | Single center | CAB, coronary artery bypass;Valve; CHD, congenital heart disease;Aortic surgery;CAB +Valve | Confusion Assessment Method of Intensive Care Unit (CAM-ICU) | 221 | 885 | Temporal validation | 100 | preoperation: age, pre-operative cerebrovascular disease, preoperative MMSE score, ASA, education degree, preoperative urea, preoperative creatinine, and preoperative album;postoperation: APACHE II, pre-operative MMSE score, CPB duration, pre-operative cerebrovascular disease, history of arrhythmia | ANN |
| 5 | Nan Lin[5] | 2024 | China | Children | Prospective Cohort Study | Single center | cardiopulmonary bypass for children diagnosed with congenital heart disease | Cornell Assessment of Pediatric Delirium (CAPD | 294 | 501 | Temporal validation | 167 | age, postoperative SpO2, lymphocyte count, diuretic use, midazolam administration | LR |
| 6 | Olga de la Varga-Martínez[6] | 2021 | Spain | Adults | Prospective Cohort Study | Multi-center | Coronary artery bypass grafting, valve replacement surgery, Mixed surgery, Others | Confusion Assessment Method-Intensive Care Unit (CAM-ICU) | 117 | 689 | Random Split Validation | 344 | MMSE score, insomnia needing medical treatment, age>65 years, low physical activity | LR |
| 7 | Ying Zhang[7] | 2024 | China | Adults | Retrospective Cohort Study | Single center | off-pump coronary artery bypass grafting (OPCABG) | Diagnostic and Statistical Manual of Mental Disorders, 5th edition | 269 | 1258 | Random Split Validation | 314 | age, tissue oxygen saturation, mean arterial pressure (MAP), carotid stenosis, the anterior-posterior diameter of the aortic sinus, ventricular septum thickness, left ventricular ejection fraction (LVEF), and Mini-Mental State Examination (MMSE) scores | LR |
| 8 | Chie Nagata[8] | 2023 | Japan | Adults | Prospective Cohort Study | Single center | coronary artery bypass graft (CABG), valve surgery, ascending aortic replacement (AAR) via a median sternotomy, minimally invasive cardiac surgery (MICS) using cardiopulmonary bypass (CPB) | the Diagnostic and Statistical Manual of Mental Disorders-5 (DSM-5) | 24 | 87 | K-fold Cross-Validation | N/A | age, use of psychotropic drugs, Mini-Cog<4, Barthel Index<100, history of stroke or cerebral hemorrhage, and eGFR<60 | XGBoost |
| 9 | Jian‑Ling Lin[9] | 2023 | China | Adults | Retrospective Cohort Study | Single center | modified triple‑branched stent graft implantation (MTBSG) surgery----aortic dissection type A | The confusion assessment method for the intensive care unit (CAM-ICU) | 220 | 692 | Bootstrap Resampling Validation | N/A | alcohol consumption, APACHE II score, postoperative serum total bilirubin, AKI Stage 3, serum IL-6, post-operative analgesic usage, ventilation duration | LR |
| 10 | Masahiro Hata[10] | 2023 | Japan | Adults | Prospective Cohort Study | Single center | CABG, coronary artery bypass graft; GR, graft replacement; Valve, Valve surgery | DSM-V criteria | 47 | 128 | Random Split Validation | 26(calculated) | EEG data | DT |
| 11 | Yanghui Xu[11] | 2022 | China | Adults | Retrospective(for model development)and Prospective (for model validation) Cohort Study | Single center | CABG(Coronary Artery Bypass Grafting), Valve replacement or shaping, Aortic valve replacement or shaping Heart tumor removal, Congenital correction | the fuzzy assessment of ICU patients (CAM-ICU) designed by Professor Ely | 405 | 838 | Random Split Validation | 449 | CPB duration, postoperative serum sodium, age, postoperative MV (machine ventilation) | LR |
| 12 | Anna Segernäs[12] | 2022 | Sweden | Adults | Prospective Cohort Study | Multi-center | coronary artery bypass graft (CABG) surgery, aortic valve replacement (AVR) surgery, mitral valve replacement (MVR) surgery or mitral plastic surgery, or combined surgery (CABG and AVR) | Nu-DESC and CAM-ICU | 47 | 218 | None | None | MMSE<27 points, A Quick Test of Cognitive Speed(AQT) color and form >70 seconds, Hospital anxiety and depression scale-depression  HAD-D>4 points, Cardiopulmonary bypass time | LR |
| 13 | Shining Cai[13] | 2022 | China | Adults | Prospective Cohort Study | Single center | cardiac surgery | Confusion Assessment Method for the ICU (CAM-ICU) | 101 | 868 | Temporal validation | 214 | Stage I prediction model(Preoperative prediction model):Age, BMI, Barthel Index for ADL, blood albumin, TBIL, positive hepatitis C virus (HCV) antibody; Stage II prediction model(prediction model based on preoperative and postoperative variables): Preoperative variable: age, blood albumin, TBIL, positive HCV antibody; Postoperative variable: operation duration, total blood loss, intraoperative ultrafiltration, APACHE II score, heart rate, MAP | LR |
| 14 | Junfeng He[14] | 2021 | China | Adults | Retrospective Cohort Study | Multi-center | type A aortic dissection treated with open-heart surgery | Confusion Assessment Method for the ICU (CAM-ICU) | 78 | 438 | Temporal validation^2^ | 30 | Smoking, Diabetes, Previous cardiovascular surgery, EF (%), Aortic block time, Acute kidney injury, Low cardiac output syndrome, Pulmonary complications | LR |
| 15 | Katarzyna Kotfis[15] | 2019 | Poland | Adults | Retrospective Cohort Study | Single center | CABG: coronary artery bypass grafting | Polish version of the CAM-ICU | 129 | 968 | Random Split Validation | Random partitioning method: not reported. | age, HbA1c, PWR (Platelet-to-WBC Ratio) | LR |
| 16 | Miarca ten Broeke[16] | 2018 | The Netherlands | Adults | Prospective Cohort Study | Single center | CABG: coronary artery bypass grafting, Valve, CABG + valve, Other | DOSS rating | 42 | 329 | None^3^ | None | Age, Comorbidity >1 , Delirium in history, SMMSE (standard mini-mental state examination) score | LR |
| 17 | Catherine C. Price[17] | 2017 | USA | Adults | Prospective Cohort Study | Single center | CABG Valve surgery Ascending aorta surgery Cardiopulmonary bypass use n (%) or mean (SD) 290 (48.8%) 386 (65.0%) 53 (8.9%) 423 (71.2%) Duration of cardiopulmonary bypass, min 108.6 (88.7) Aorta cross clamp use | Confusion Assessment Method for ICU delirium (CAM-ICU) and the Richmond Agitation and Sedation Scale (RASS) | 137 | 594 | None | None | Age, Sex, History of, COPD, Duration of cardiopulmonary bypass, Working Memory, Delayed Recall | LR |
| 18 | Sandra Koster[18] | 2012 | the Netherlands | Adults | Prospective Cohort Study | Single center | CABG, Valve, CABG + valve | the DOS scale | 52 | 300 | None^3^ | None | a higher Euroscore(European Heart Surgery Risk Assessment), age (≥70 years), cognitive impairment, number of comorbidities, history of delirium, alcohol use, type of surgery | LR |
| 19 | Qiuying Li[19] | 2024 | China | Adults | Retrospective Cohort Study | Single center | cardiac valve surgery with CPB | Richmond Assessment Sedation Scale (RASS score) and Confusion Assessment Method (CAM) | 141 | 507 | Random Split Validation | 102 | In the full feature dataset:  Preoperative Information:  Female,Education score ,Age,Height,  Weight,Alcohol abuse,Smoke abuse,Coronary heart disease,Cerebral infarction,Diabetes,Hypertension,LVEF.  Intraoperative Information:  CPB duration,ACC duration,Anesthesia duration  Postoperative Information:  IABP employ,ECMO employ,WBC,NEUT,LY,BUN,TBLL,Serum creatinine,Serum albumin,PH,PaCO2,PaO2,Na,K,Glu,Pain score | RF |
| 20 | Yu Tian[20] | 2023 | China | Adults | Retrospective Cohort Study | Single center | open cardiac surgery (including coronary artery bypass grafting (CABG), valve surgery, great vessel operations, congenital heart disease repair, cardiac tumor surgery and combined surgery) | both medication records and nursing records(AD) | 2085 | 57180 | Random Split Validation | 11436 | preoperative LVEF ≤ 45%, serum creatinine>100 µmol/L, emergency surgery, coronary artery disease, hemorrhage volume>600 mL, intraoperative platelet or plasma use, postoperative LVEF ≤ 45% | LR |
| 21 | Xiao Li[21] | 2022 | China | Adults | Prospective Cohort Study | Single center | CABG, Valve surgeries, Cardiac tumor resection , Aortic arch repairs, Others, Combined cardiac surgeries | RASS and CAMICU | 242 | 379 | Random Split Validation | 132 | Age, duration of CPB time, CD4 + T cell count, CD4/CD8 ratio | LR |
| 22 | Terezia B. Andrási[22] | 2022 | Germany | Adults | Retrospective Cohort Study | Single center | isolated coronary artery bypass grafting (CABG) , valve surgery and/or ascending aortic surgery with or without CABG | German version of the Confusion Assessment Method for Intensive Care Unit test | 93 | 300 | K-fold Cross-Validation | N/A | age, CPB time, MV(machine ventilation) time, fresh frozen plasma transfusion, postoperative atrial fibrillation | LR |
| 23 | James L. Rudolph[23] | 2009 | USA | Adults | Prospective Cohort Study | Multi-center | coronary artery bypass graft (CABG), mitral or aortic valve replacement or repair (valve), and combined CABG-Valve | CAM/CAMICU | 111 | 231 | External Validation | 109 | prior stroke or transient ischemic attack (TIA), Mini Mental State Examination (MMSE) score, abnormal serum albumin, the Geriatric Depression Scale (GDS) score | LR |
| 24 | Ya-peng Wang[24] | 2022 | China | Adults | Prospective Cohort Study | Single center | Valve surgery, CABG, Valve + CABG, Valve + Maze operation, Aortic surgery, Congenital heart disease | Confusion Assessment Method for the ICU (CAM-ICU) | 28 | 232 | Bootstrap Resampling Validation | N/A | postoperative lactate levels, maximum temperature, cardiopulmonary bypass(CP) time | LR |
| 25 | Tan Yang[25] | 2023 | China | Adults | Retrospective Cohort Study | Multi-center | ONCAB: on-pump coronary artery bypass; OPCAB: off-pump coronary artery bypass;VR and vr: Valve Replacement and valve repair;Repair of ASD or VSD (ASD or VSD: Atrial Septal Defect or Ventricular Septal Defect);AR and others (Aortic Replacement) | diagnostic protocols for delirium features based on the Diagnostic and Statistical Manual of Mental Disorders, Fifth Edition (DSM-5) | 105 | 367 | K-fold Cross-Validation | N/A | age, smoking, alcohol consumption, visual impairment, hearing impairment, educational level, history of hypertension, history of diabetes, history of atrial fibrillation, carotid artery stenosis, haemoglobin, albumin levels, serum creatinine, CPB time, aortic clamping time, anaesthesia time, operation duration, postoperative sedation, ICU stay, length of hospital stay | KNN |
| 26 | Monika Sadlonova[26] | 2023 | Germany | Adults | Prospective Cohort Study | Single center | CABG: coronary bypass artery grafting, Heart valve surgery, Combined CABG and heart valve, Cardiopulmonary bypass | CAM/CAMICU | 106 | 504 | Random Split Validation | 151(calculated) | Delirium Risk Screening Questionnaire (DRSQ), Montreal Cognitive Assessment (MoCA), Trail Making Test-B (TMTB) | LASSO |
| 27 | Hani Nabeel Mufti[27] | 2019 | Saudi Arabia | Adults | Retrospective Cohort Study | Single center | Coronary artery bypass graft, Aortic valve replacement, Mitral valve surgery, CABG+AVR, CABG+MV surgery (CABG+AVR: coronary artery bypass graft + aortic valve replacement, CABG+MV: coronary artery bypass graft + mitral valve.) | CAMICU | 507 | 5584 | Random Split Validation | 1117 | Age, Mechanical ventilation>24 hours, Preoperative creatinine clearance, Length of stay in the ICU, surgical type(Procedure other than isolated CABG), Blood product within 48 hours, Intraoperative TEE, Intraoperative inotropes, COPD, CVD, DM, Frail, History of turn down, EF categories, Gender, Aortic stenosis, Mitral insufficiency, Postoperative arrhythmias | ANN |
| 28 | Linda Lapp[28] | 2022 | UK | Adults | Retrospective Cohort Study | Database | coronary artery bypass graft (CABG), valve, combined CABG and valve surgery | CAM-ICU | 414(calculated) | 3322 | Random Split Validation | 762 | Static pre-operative variables from CaTHI database: Patient characteristics: Age, sex, type II diabetes, BMI, smoking status;Cardiac preoperative variables: Congestive cardiac failure, previous myocardial infarction, active endocarditis, hypertension history, NYHA grade, angina status, rhythm, left ventricular function, left main stem, extracardiac arteriopathy, logistic EuroSCORE;Non-cardiac preoperative variables: Neurological dysfunction, pulmonary disease, preoperative creatinine, renal impairment;Surgical variables: Surgical priority, critical preoperative state, procedure, previous cardiac surgery, previous percutaneous coronary intervention.  Dynamic ICU variables from Centricity Critical Care database: laboratory variables(Minimum, maximum, first, last): Arterial base excess, arterial haematocrit, bicarbonate, c-reactive protein, creatinine, daily fluid balance, haemoglobin, hydrogen ion, lactate, potassium, sodium, urea, urine;Binary variables(yes/no): CAM-ICU; Medicines: Dobutamine, dopamine, noradrenaline, vasopressin | SVM |

Note.1.None:Lacking a validation set, thus, no validation data.,2:Study 14: Temporal validation was attempted, but no model performance data were reported owing to a small sample size,3:Studies 16 and 18 did not validate the new model on an independently created dataset; consequently, no validation set data are available.

Abbreviations:N/A:Not Applicable,LR: Logistic Regression,LGBM: Light Gradient Boosting Machine,ANN: Artificial Neural Network,XGBoost: Extreme Gradient Boosting,DT: Decision Tree,RF:Random Forest Classifier,KNN: K-Nearest Neighbors,LASSO: Least Absolute Shrinkage and Selection Operator,SVM: Support Vector Machine

1. Hatano Y, Narumoto J, Shibata K, Matsuoka T, Taniguchi S, Hata Y, et al. White-matter hyperintensities predict delirium after cardiac surgery. Am J Geriatr Psychiatry. 2013 Oct;21(10):938-45. PMID: 24029014. doi: 10.1016/j.jagp.2013.01.061.
2. Mao D, Fu L, Zhang W. Risk Factors and Nomogram Model of Postoperative Delirium in Children with Congenital Heart Disease: A Single-Center Prospective Study. Pediatr Cardiol. 2024 Jan;45(1):68-80. PMID: 37741935. doi: 10.1007/s00246-023-03297-5.
3. Han C, Kim HI, Soh S, Choi JW, Song JW, Yoon D. Machine learning with clinical and intraoperative biosignal data for predicting postoperative delirium after cardiac surgery. iScience. 2024 Jun 21;27(6):109932. PMID: 38799563. doi: 10.1016/j.isci.2024.109932.
4. Zhao X, Li J, Xie X, Fang Z, Feng Y, Zhong Y, et al. Online interpretable dynamic prediction models for postoperative delirium after cardiac surgery under cardiopulmonary bypass developed based on machine learning algorithms: A retrospective cohort study. J Psychosom Res. 2024 Jan;176:111553. PMID: 37995429. doi: 10.1016/j.jpsychores.2023.111553.
5. Lin N, Lv M, Li S, Xiang Y, Li J, Xu H. A nomogram for predicting postoperative delirium in pediatric patients following cardiopulmonary bypass: A prospective observational study. Intensive Crit Care Nurs. 2024 Aug;83:103717. PMID: 38692080. doi: 10.1016/j.iccn.2024.103717.
6. de la Varga-Martínez O, Gómez-Pesquera E, Muñoz-Moreno MF, Marcos-Vidal JM, López-Gómez A, Rodenas-Gómez F, et al. Development and validation of a delirium risk prediction preoperative model for cardiac surgery patients (DELIPRECAS): An observational multicentre study. J Clin Anesth. 2021 May;69:110158. PMID: 33296785. doi: 10.1016/j.jclinane.2020.110158.
7. Zhang Y, Ren M, Zhai W, Han J, Guo Z. Construction and validation of a risk prediction model for postoperative delirium in patients with off‑pump coronary artery bypass grafting. J Thorac Dis. 2024 Jun 30;16(6):3944-55. PMID: 38983165. doi: 10.21037/jtd-24-578.
8. Nagata C, Hata M, Miyazaki Y, Masuda H, Wada T, Kimura T, et al. Development of postoperative delirium prediction models in patients undergoing cardiovascular surgery using machine learning algorithms. Sci Rep. 2023 Nov 30;13(1):21090. PMID: 38036664. doi: 10.1038/s41598-023-48418-5.
9. Lin JL, Zheng GZ, Chen LW, Luo ZR. A nomogram model for assessing predictors and prognosis of postoperative delirium in patients receiving acute type A aortic dissection surgery. BMC Cardiovasc Disord. 2023 Feb 7;23(1):72. PMID: 36750929. doi: 10.1186/s12872-023-03111-3.
10. Hata M, Miyazaki Y, Nagata C, Masuda H, Wada T, Takahashi S, et al. Predicting postoperative delirium after cardiovascular surgeries from preoperative portable electroencephalography oscillations. Front Psychiatry. 2023;14:1287607. PMID: 38034919. doi: 10.3389/fpsyt.2023.1287607.
11. Xu Y, Meng Y, Qian X, Wu H, Liu Y, Ji P, et al. Prediction model for delirium in patients with cardiovascular surgery: development and validation. J Cardiothorac Surg. 2022 Oct 1;17(1):247. PMID: 36183105. doi: 10.1186/s13019-022-02005-3.
12. Segernäs A, Skoog J, Ahlgren Andersson E, Almerud Österberg S, Thulesius H, Zachrisson H. Prediction of Postoperative Delirium After Cardiac Surgery with A Quick Test of Cognitive Speed, Mini-Mental State Examination and Hospital Anxiety and Depression Scale. Clin Interv Aging. 2022;17:359-68. PMID: 35400995. doi: 10.2147/cia.S350195.
13. Cai S, Cui H, Pan W, Li J, Lin X, Zhang Y. Two-stage prediction model for postoperative delirium in patients in the intensive care unit after cardiac surgery. Eur J Cardiothorac Surg. 2022 Dec 2;63(1). PMID: 36579859. doi: 10.1093/ejcts/ezac573.
14. He J, Ling Q, Chen Y. Construction and Application of a Model for Predicting the Risk of Delirium in Postoperative Patients With Type a Aortic Dissection. Front Surg. 2021;8:772675. PMID: 34869569. doi: 10.3389/fsurg.2021.772675.
15. Kotfis K, Ślozowska J, Safranow K, Szylińska A, Listewnik M. The Practical Use of White Cell Inflammatory Biomarkers in Prediction of Postoperative Delirium after Cardiac Surgery. Brain Sci. 2019 Nov 2;9(11). PMID: 31684066. doi: 10.3390/brainsci9110308.
16. Ten Broeke M, Koster S, Konings T, Hensens AG, van der Palen J. Can we predict a delirium after cardiac surgery? A validation study of a delirium risk checklist. Eur J Cardiovasc Nurs. 2018 Mar;17(3):255-61. PMID: 28980478. doi: 10.1177/1474515117733365.
17. Price CC, Garvan C, Hizel LP, Lopez MG, Billings FTt. Delayed Recall and Working Memory MMSE Domains Predict Delirium following Cardiac Surgery. J Alzheimers Dis. 2017;59(3):1027-35. PMID: 28697572. doi: 10.3233/jad-170380.
18. Koster S, Hensens AG, Schuurmans MJ, van der Palen J. Prediction of delirium after cardiac surgery and the use of a risk checklist. Eur J Cardiovasc Nurs. 2013 Jun;12(3):284-92. PMID: 22694810. doi: 10.1177/1474515112450244.
19. Li Q, Li J, Chen J, Zhao X, Zhuang J, Zhong G, et al. A machine learning-based prediction model for postoperative delirium in cardiac valve surgery using electronic health records. BMC Cardiovasc Disord. 2024 Jan 18;24(1):56. PMID: 38238677. doi: 10.1186/s12872-024-03723-3.
20. Tian Y, Ji B, Diao X, Wang C, Wang W, Gao Y, et al. Dynamic predictive scores for cardiac surgery-associated agitated delirium: a single-center retrospective observational study. J Cardiothorac Surg. 2023 Jul 6;18(1):219. PMID: 37415226. doi: 10.1186/s13019-023-02339-6.
21. Li X, Cheng W, Zhang J, Li D, Wang F, Cui N. Early alteration of peripheral blood lymphocyte subsets as a risk factor for delirium in critically ill patients after cardiac surgery: A prospective observational study. Front Aging Neurosci. 2022;14:950188. PMID: 36118695. doi: 10.3389/fnagi.2022.950188.
22. Andrási TB, Talipov I, Dinges G, Arndt C, Rastan AJ. Risk factors for postoperative delirium after cardiac surgical procedures with cardioplegic arrest. Eur J Cardiothorac Surg. 2022 Jun 15;62(1). PMID: 35037042. doi: 10.1093/ejcts/ezab570.
23. Rudolph JL, Jones RN, Levkoff SE, Rockett C, Inouye SK, Sellke FW, et al. Derivation and validation of a preoperative prediction rule for delirium after cardiac surgery. Circulation. 2009 Jan 20;119(2):229-36. PMID: 19118253. doi: 10.1161/circulationaha.108.795260.
24. Wang YP, Shen BB, Zhu CC, Li L, Lu S, Wang DJ, et al. Unveiling the nexus of postoperative fever and delirium in cardiac surgery: identifying predictors for enhanced patient care. Front Cardiovasc Med. 2023;10:1237055. PMID: 38028495. doi: 10.3389/fcvm.2023.1237055.
25. Yang T, Yang H, Liu Y, Liu X, Ding YJ, Li R, et al. Postoperative delirium prediction after cardiac surgery using machine learning models. Comput Biol Med. 2024 Feb;169:107818. PMID: 38134752. doi: 10.1016/j.compbiomed.2023.107818.
26. Sadlonova M, Hansen N, Esselmann H, Celano CM, Derad C, Asendorf T, et al. Preoperative Delirium Risk Screening in Patients Undergoing a Cardiac Surgery: Results from the Prospective Observational FINDERI Study. Am J Geriatr Psychiatry. 2024 Jul;32(7):835-51. PMID: 38228452. doi: 10.1016/j.jagp.2023.12.017.
27. Mufti HN, Hirsch GM, Abidi SR, Abidi SSR. Exploiting Machine Learning Algorithms and Methods for the Prediction of Agitated Delirium After Cardiac Surgery: Models Development and Validation Study. JMIR Med Inform. 2019 Oct 23;7(4):e14993. PMID: 31558433. doi: 10.2196/14993.
28. Lapp L, Roper, M., Kavanagh, K., & Schraag, S. Predicting the onset of delirium on hourly basis in an intensive care unit following cardiac surgery. IEEE 35th International Symposium on Computer-Based Medical Systems (CBMS). 2022:234-9. doi: 10.1109/cbms55023.2022.00048.
