# Supplementary material for: The Predictive Value of Machine Learning for Postoperative Delirium in Cardiac Surgery: Systematic Review and Meta-Analysis
Source: J Med Internet Res. 2026 Feb 23;28:e72304. doi: 10.2196/72304 (PMC12928544; doi:10.2196/72304)
Supplement: Multimedia Appendix 3 [file jmir-v28-e72304-s003.docx]

**Table S3** Modeling variables in included studies.

| No. | Factors | Times |
| --- | --- | --- |
|  | Preoperative period |  |
|  | Basic information |  |
| 1 | Age | 19 |
| 2 | Gender | 4 |
| 3 | Smoking | 4 |
| 4 | Alcohol consumption | 3 |
| 5 | Education degree | 3 |
| 6 | BMI | 2 |
| 7 | Height | 1 |
| 8 | Weight | 1 |
|  | Physical Activity Capacity |  |
| 9 | Barthel Index | 2 |
| 10 | Katz grade 4 | 1 |
| 11 | Frail | 1 |
| 12 | Low physical activity | 1 |
|  | Comorbidity |  |
| 13 | Diabetes | 5 |
| 14 | History of stroke or cerebral hemorrhage or TIA | 5 |
| 15 | Comorbidity | 2 |
| 16 | History of hypertension | 3 |
| 17 | Pre-operative history of Coronary heart disease | 3 |
| 18 | Delirium in history | 2 |
| 19 | Depression(GDS score) | 2 |
| 20 | Insomnia needing medical treatment | 1 |
| 19 | Neurological dysfunction | 1 |
| 21 | Visual impairment | 1 |
| 22 | Hearing impairment | 1 |
| 23 | COPD | 1 |
| 24 | Angina status | 1 |
| 25 | Congestive cardiac failure | 1 |
| 26 | History of arrhythmia | 1 |
| 27 | Previous myocardial infarction | 1 |
| 28 | Active endocarditis | 1 |
| 29 | History of atrial fibrillation | 1 |
| 30 | Extracardiac arteriopathy | 1 |
| 31 | Renal impairment | 1 |
|  | Past Medical History |  |
| 32 | Previous cardiac and cardiovascular surgery | 2 |
| 33 | Heart rate | 2 |
| 34 | Use of psychotropic drugs | 1 |
| 35 | History of turn down | 1 |
| 36 | Previous percutaneous coronary intervention | 1 |
|  | Laboratory Examinations |  |
| 37 | Creatinine | 8 |
| 38 | Albumin | 5 |
| 39 | Preoperative TBIL | 2 |
| 40 | Positive hepatitis C virus antibody | 2 |
| 41 | eGFR | 2 |
| 42 | HbA1c | 1 |
| 43 | PWR (Platelet-to-WBC Ratio) | 1 |
| 44 | Urea | 1 |
| 45 | CD4 + T cell count | 1 |
| 46 | CD4/CD8 ratio | 1 |
| 47 | Serum IL-6 | 1 |
| 48 | Daily fluid balance | 1 |
| 49 | T3 | 1 |
|  | Imaging Examinations |  |
| 50 | Left ventricular ejection fraction | 6 |
| 51 | Carotid stenosis | 3 |
| 52 | EEG data | 1 |
| 53 | DWMH | 1 |
| 54 | The anterior-posterior diameter of the aortic sinus | 1 |
| 55 | Ventricular septum thickness | 1 |
|  | Rating |  |
| 56 | APACHE II | 3 |
| 57 | A higher Euroscore | 2 |
| 58 | ASA | 1 |
| 59 | Critical preoperative state | 1 |
| 60 | NYHA grade | 1 |
|  | Cognitive Function-Related Items |  |
| 61 | MMSE | 7 |
| 62 | Cognitive impairment | 1 |
| 63 | Mini-Cog < 4 | 1 |
| 64 | Montreal Cognitive Assessment | 1 |
| 65 | Working Memory | 1 |
| 66 | Delayed Recall | 1 |
| 67 | Trail Making Test-B | 1 |
| 68 | Delirium Risk Screening Questionnaire | 1 |
|  | Intraoperative period |  |
|  | Surgical priority |  |
| 69 | Emergency surgery | 3 |
| 70 | Surgical priority | 1 |
|  | Type of surgery |  |
| 71 | Type of surgery | 4 |
| 72 | Procedure other than an isolated CABG | 1 |
| 73 | Mitral insufficiency | 1 |
| 74 | Left main stem | 1 |
|  | Time |  |
| 75 | CPB duration | 9 |
| 76 | Duration of surgery | 4 |
| 77 | Aortic block time | 3 |
| 78 | Anesthesia duration | 2 |
|  | Intraoperative Procedures |  |
| 79 | Intraoperative inotropes | 2 |
| 80 | Intraoperative ultrafiltration | 1 |
| 81 | Intraoperative TEE | 1 |
| 82 | Delayed chest closure | 1 |
| 83 | Use of mannitol | 1 |
| 84 | Intraoperative platelet or plasma use | 1 |
|  | Intraoperative Monitoring Indicators |  |
| 85 | ARV of mean arterial pressure | 3 |
| 86 | Total blood loss | 2 |
| 87 | Duration of low BIS | 2 |
| 88 | AUC of PP < 60 mmHg | 1 |
| 89 | Average CI | 1 |
| 90 | AQT color and form >70 seconds | 1 |
|  | Postoperative period |  |
|  | Postoperative Evaluation and Treatment |  |
| 91 | Sedation and Sedative Drugs’ dosage | 4 |
| 92 | Mechanical ventilation time | 3 |
| 93 | Blood product transfusion in the first 48 h | 1 |
| 94 | Non-invasive ventilation after extubation | 1 |
| 95 | Pain score | 1 |
| 96 | Postoperative analgesic usage | 1 |
| 97 | Diuretic use | 1 |
| 98 | CAM-ICU | 1 |
| 99 | Fresh frozen plasma transfusion | 1 |
|  | Postoperative Monitoring |  |
| 100 | Postoperative SpO2 | 2 |
| 101 | Postoperative lactate levels | 2 |
| 102 | rSO2 | 2 |
| 103 | Haemoglobin | 2 |
| 104 | Lymphocyte count | 2 |
| 105 | Potassium | 2 |
| 106 | Urea | 2 |
| 107 | IABP employ | 1 |
| 108 | ECMO employ | 1 |
| 109 | C-reactive protein | 1 |
| 110 | Arterial base excess | 1 |
| 111 | Arterial haematocrit | 1 |
| 112 | Duration of SR > 1% | 1 |
| 113 | Postoperative serum total bilirubin | 1 |
| 114 | WBC | 1 |
| 115 | NEUT | 1 |
| 116 | Serum albumin | 1 |
| 117 | Sodium | 1 |
| 118 | Hydrogenion | 1 |
| 119 | Bicarbonate | 1 |
| 120 | Urine | 1 |
| 121 | PaCO2 | 1 |
| 122 | PaO2 | 1 |
| 123 | PH | 1 |
| 124 | Na | 1 |
| 125 | Glu | 1 |
|  | Postoperative Complications |  |
| 126 | Pulmonary complications | 3 |
| 127 | AKI | 2 |
| 128 | Elevated temperature | 2 |
| 129 | Low cardiac output syndrome | 1 |
| 130 | Postoperative arrhythmias | 1 |
| 131 | Postoperative atrial fibrillation | 1 |
| 132 | Length of hospital stay | 1 |

Abbreviations: BMI: Body Mass Index; TIA: Transient Ischemic Attack; CVD: Cardiovascular Disease; GDS score: Geriatric Depression Scale score; COPD: Chronic Obstructive Pulmonary Disease; TBIL: Total Bilirubin; eGFR: Estimated Glomerular Filtration Rate; HbA1c: Hemoglobin A1c; EEG data: Electroencephalogram data; DWMH: Deep White Matter Hyperintensity; APACHE II: Acute Physiology and Chronic Health Evaluation II; ASA: American Society of Anesthesiologists Physical Status Classification; NYHA grade: New York Heart Association Functional Classification; MMSE: Mini-Mental State Examination; Mini-Cog: Mini-Cognitive Assessment; CABG: Coronary Artery Bypass Grafting; CPB: Cardiopulmonary Bypass; TEE: Transesophageal Echocardiography; ARV: Average Real Variability; PP: Pulse Pressure; CI: Cardiac Index; AQT: A Quick Test of Cognitive Speed; CAM-ICU: Confusion Assessment Method for the Intensive Care Unit; SpO₂: Peripheral Capillary Oxygen Saturation; rSO₂: Regional Oxygen Saturation; IABP: Intra-Aortic Balloon Pump; ECMO: Extracorporeal Membrane Oxygenation; WBC: White Blood Cell; NEUT: Neutrophil; PH: Potential of Hydrogen; AKI: Acute Kidney Injury; ICU: Intensive Care Unit
